# Parental food provisioning and nestling growth under *Philornis downsi* parasitism in the Galapagos Green Warbler-Finch, classified as ‘vulnerable’ by the IUCN

**DOI:** 10.1007/s10336-023-02049-9

**Published:** 2023-02-18

**Authors:** Courtney L. Pike, Barbara Kofler, Heinz Richner, Sabine Tebbich

**Affiliations:** 1 Department of Behavioral and Cognitive Biology, University of Vienna, Vienna, Vienna Austria; 2 Department of Biology, University of Bern, Bern, Bern Switzerland

**Keywords:** Nest parasite, Parental care, Nestling growth, Host defense, Galapagos Islands, *Philornis downsi*

## Abstract

In the Galapagos Islands, many endemic landbird populations are declining due to habitat degradation, food availability, introduced species and other factors. Given nestlings typically lack efficient defense mechanisms against parasites, hematophagous ectoparasites such as the larvae of the introduced Avian Vampire Fly, *Philornis downsi*, can impose high brood mortality and cause threatening population declines in Darwin finches and other landbirds. Here, we assess whether the food compensation hypothesis (i.e., the parents’ potential to compensate for deleterious parasite effects via increased food provisioning) applies to the Green Warbler-Finch. We differentiated nests with low or high infestation levels by *P*. *downsi* and quantified food provisioning rates of male and female parents, time females spent brooding nestlings, and nestling growth. Male provisioning rates, total provisioning rates and female brooding time did not significantly vary in relation to infestation levels, nor by the number of nestlings. Opposed to the predictions of the food compensation hypothesis, females showed significantly reduced provisioning rates at high infestation levels. Nestling body mass was significantly lower and there was a reduction of skeletal growth, although not significantly, in highly infested nests. The females’ response to high infestation may be due to parasites directly attacking and weakening brooding females, or else that females actively reduce current reproductive effort in favor of future reproduction. This life-history trade-off may be typical for Darwin finches and many tropical birds with long lifespans and therefore high residual reproductive value. Conservation strategies may not build on the potential for parental food compensation by this species.

## Abstract (de)

**Elterliche Nahrungsversorgung und Nestlingswachstum beim durch die IUCN als „gefährdet “ eingestuften Waldsänger-Darwinfink bei Befall durch die parasitische invasive Vampirfliege** ***Philornis downsi***

Auf den Galapagos-Inseln sind auf Grund von Habitatstörungen, eingeschleppten und invasiven Organismen und anderen Faktoren viele Vogelarten und Populationen in ihrer Existenz bedroht. Beispielsweise führen die hämatophagen Larven der eingeführten Vampirfliege *Philornis downsi* zu einer hohen Brutsterblichkeit unter den endemischen Darwinfinken, insbesondere da bei Nestlingen die Immunabwehr noch schwach und unvollständig ausgeprägt ist. Hier wird untersucht, ob brütende Waldsänger-Darwinfinken *Certhidea olivacea* in der Lage sind, die für das Nestlingswachstum schädlichen Parasiteneffekte durch eine erhöhte Fütterungsrate zu kompensieren (Nahrungskompensations-Hypothese). Entgegen dieser Hypothese zeigte sich zwischen Nestern mit schwachem und starkem Parasitenbefall kein signifikanter Unterschied sowohl in der Gesamtfütterungsrate der Nestlinge als auch in der Anwesenheitsdauer der Weibchen im Nest. Entsprechend waren Nestlinge in stark befallenen Nestern leichter und im Skelettwachstum eingeschränkt. Weibchen in stark befallenen Nestern zeigten sogar signifikant geringere Fütterungsraten, weil möglicherweise Parasitenlarven auch brütende Weibchen befallen und schwächen. Möglich ist auch, dass Weibchen ihre derzeitigen Reproduktionsanstrengungen zugunsten einer zukünftigen Reproduktion aktiv reduzieren, wie durch die Life-history-Theorie vorausgesagt wird. Dieser Life-history Trade-off kann insbesondere bei Darwinfinken und tropischen Vogelarten, auf Grund ihrer relativ langen Lebensdauer und demzufolge einem relativ hohen zukünftigen Reproduktionswert, erwartet werden. Maßnahmen zum Schutz von Darwinfinken sollten deshalb ihr beschränktes Potenzial zur Nahrungskompensation miteinbeziehen.

## Introduction

Birds are hosts of many types of parasites, including nest ectoparasites (for reviews, see e.g., Loye and Zuk 1991; Crompton 1997; Janovy 1997; López-Rull and Macías Garcia 2015). Nestlings of altricial birds are ideal hosts for ectoparasites since they are left unattended in the nest for many hours each day and have limited defenses against parasites, due to their reduced mobility and their still naïve immune functioning. Selection will thus favor parental control of parasite numbers or compensation of parasite effects on nestlings (e.g., Tschirren et al. 2009; López-Rull and Macías Garcia 2015). However, parental compensation via a higher food supply to nestlings, or other behaviors, will increase current reproductive effort, but at the expense of reducing residual reproductive value (Perrin et al. 1996), a life-history trade-off experimentally demonstrated in many studies (e.g., Richner and Tripet 1999). Higher food provisioning allows nestlings to allocate more resources to blood and tissue replacement and growth (Mason 1944; Johnson and Albrecht 1993; Morrison and Johnson 2002). The extent of parental compensation may be influenced by environmental conditions and the amount or quality of available food (Tremblay et al. 2005), among other factors. However, ectoparasites may also exploit and weaken the brooding female, which may then decrease food provisioning rates and presence and direct care of nestlings (Koop et al. 2013; Knutie et al. 2016).

In the Galapagos Islands, Ecuador, one of the major threats to many landbird species including most Darwin finches is the invasive Avian Vampire Fly, *Philornis downsi* (Diptera: Muscidae). Female flies lay eggs in bird nests where subsequently the hematophagous larvae feed on blood and tissue of nestlings (Fessl et al. 2006b). First reports of the fly date back to the 1960’s (Causton et al. 2006) and thus the fly is assumed to have recently invaded the Galapagos archipelago. It currently exploits at least 21 native and endemic Galapagos landbird species (Fessl et al. 2018; Anchundia and Fessl 2020; Coloma et al. 2020), several of which are currently classified by the IUCN as vulnerable or critically endangered (IUCN 2022). *Philornis downsi* can cause detrimental effects including blood loss, poor growth, deformed nasal cavities that interfere with sexual selection (Kleindorfer et al. 2019), and up to 100% brood mortality (reviewed in Kleindorfer and Dudaniec 2016). Lower breeding success caused by *P*. *downsi* parasitism has been documented for the majority of Darwin’s finch species and the Little Vermilion Flycatcher (*Pyrocephalus nanus*) (as reviewed in Fessl et al. 2018; Cimadom and Tebbich 2021; Mosquera et al. 2022). Evidence for parental compensation via an increase in care and food provisioning to nestlings in Galapagos bird species is mixed: Knutie et al. (2016) showed that Galapagos Mockingbird (*Mimus parvulus*) parents can compensate for *P*. *downsi* parasitism, but this compensation was not found in another study (McNew et al. 2019), suggesting that parental compensation is a flexible response in this species. No compensation via increased parental provisioning rate was found in the Small Ground Finch (*Geospiza fuliginosa*), Medium Ground Finch (*Geospiza fortis*) (O’Connor et al. 2014; Knutie et al. 2016) or the Small Tree Finch (*Camarhynchus parvulus*) (Heyer et al. 2021). In a comparative study, Green Warbler-Finches (*Certhidea olivacea*) showed both a significantly lower infestation intensity and higher breeding success than sympatric Small Tree Finches (*Camarhynchus parvulus*) (Cimadom et al. 2014), which could be due either to lower parasite numbers or parental compensation, or both.

Many tree finch species in Galapagos, including two critically endangered species, the Mangrove Finch (*Camarhynchus heliobates*) and the Medium Tree Finch (*Camarhynchus pauper*), are in decline in part due to *P*. *downsi* parasitism (Fessl et al. 2010, 2017; Dvorak et al. 2012, 2017; Cimadom et al. 2014). Populations of the Green Warbler-Finch (*Certhidia olivacea*) have continued to decline, including in its small habitat range in Santa Cruz Island, as shown by close monitoring of populations since 1997 (Dvorak et al. 2012; Cimadom et al. 2014; Fessl et al. 2017). Understanding behavioral adaptations of avian hosts to *P*. *downsi* parasites, including the parents’ ability to compensate for effects of parasites, is critical for the choice and allocation of conservation efforts.

In this study, we evaluated whether male and female Green Warbler-Finches compensate for *P*. *downsi* parasitism via higher provisioning rates and feeding efforts. We assessed infestation levels in Warbler-Finch nests and for data analysis grouped nests in low and high infestation categories, respectively. Our working hypothesis of parental compensation predicts that one or both parents increase the rate of food provisioning under high parasite infestation intensity, and that consequently nestlings of the high and low infestation categories should show similar growth and development. Knowledge of parental compensation behavior and efficiency is relevant for informing conservation efforts and strategies.

## Methods

### Study site

The study took place within the Galapagos National Park protected areas at Los Gemelos (0° 37′ 50″ S, 90° 23′ 25″ W, altitude 569–625 m a.s.l.) on Santa Cruz Island in the Galapagos Islands, Ecuador. The site consists of a 0.33 km^2^ area in a cloud forest habitat dominated by the endemic *Scalesia pedunculata* and includes other characteristic plant species such as the cat’s claw (*Zanthoxylum fagara*), ferns, and the endemic Galapagos guava tree (*Psidium galapagium*) (CLP, pers. obs.; McMullen 1999). Since 2012, this area has also undergone long-term management for the invasive Mysore raspberry (*Rubus niveus*) (Cimadom et al. 2019).

### Nest monitoring and treatment

From February to mid-April 2021, Green Warbler-Finch nests (*n* = 26) were monitored for activity every two to three days during incubation. Incubating nests close to hatching were monitored every one to two days for recording the precise hatch date. Nests located in the canopy of *Scalesia* trees at a height of 1.5 to 5.4 m were monitored for parental activity during incubation and using an endoscopic camera attached to a long carbon fiber pole to visually inspect nestlings in the nest after hatching. Eight nests were treated against parasites by manual injection with 5 mL of a 0.5% permethrin solution (Permacap CS) (for details of this method see Cimadom et al. 2019), applied with a 5 mL plastic syringe inserted into the base of the nest from the outside. Nests were treated within three days before or after the nestlings hatched. Hatching date was recorded as the date when the first egg hatched and was confirmed by visual observation via the camera monitor. Nestlings on hatch date were recorded as zero days old.

### Feeding rate observations

Each nest was observed for a duration of 60 min between 6:30 a.m. and 10:00 a.m. when the nestlings were five to six days old. The number of feeding visits by the male and female were recorded, along with the duration the female spent inside the nest. Observations were not conducted in rainy weather. Observers kept a minimum distance of four meters from the nest as to not disturb the parents, a distance also used in other studies (e.g. Heyer et al. 2021).

Data recorded at each nest were standardized following the methodology in Heyer et al. (2021). Briefly, a *feeding visit* was defined as a ‘visually confirmed insertion of food into a nestling's beak, and in cases the insertion could not be seen, a parent coming to the nest with food and leaning inside the nest in a feeding-like behavior’. For data analysis, *total provisioning rate* per nest was defined as the summed number of feeding visits per hour by both parents plus the male passing food to the female. Since males sometimes give the food to the brooding female who may then feed the nestlings, we defined *male provisioning rate* as the number of feeding visits per hour to nestlings by the male plus the feedings delivered to the female. Thus, male provisioning rates reflect total male parental effort. In principle, it is irrelevant whether food brought by the male is consumed by the female or given to the nestlings, since in the first case the female can bring more of the food items caught by herself to nestlings instead of consuming them for her own maintenance. *Female provisioning rate* was defined as the number of direct feeding visits per hour to nestlings by the female parent. *Female nest attention* was defined as the minutes per hour a female spent sitting in the nest during the observation period. Nests that were no longer active were collected, dismantled in the laboratory, and the number of *P*. *downsi* larvae and pupae were counted.

### Nestling measurements

We collected measurements on 5 to 6-day-old nestlings. To limit disturbance time to under 15 min, maximally two nestlings per nest were briefly removed from the nest. We recorded tarsus length (tarso-metatarsus in mm) of the right leg and nestling mass to the nearest 0.01 g using an electronic scale. We additionally examined each nestling for signs of *P*. *downsi* parasitism, such as larvae clinging to the body and nares or typical body wounds caused by blood-sucking larvae. All nestling manipulation was done either directly after the feeding observation, or a day before or after observation as to not disturb the natural behavior of the parents and nestlings on observation day.

### Statistical analysis

For the analysis, we grouped nests by infestation level, based on the number of *P*. *downsi* larvae or pupae found after nest collection. The “low infestation” group included nests with 0–3 parasites and the “high infestation” group included nests with 5–65 parasites. Three nests not treated with permethrin had naturally very few parasites (0, 1, and 2, respectively) and hence were added to the “low infestation group”. Two nests that were treated still had 7 and 10 parasites and thus were added to the “high infestation group”. Infestation level with *P. downsi* was significantly higher (*W* = 144, *p* < 0.001) in nests in the high infestation group than in nests in the low infestation group. Mean infestation intensity per nestling was more than 15-fold higher in the “high infestation” group (low infestation: mean = 0.59 ± SD 0.533, median = 0.50, *n* = 9; high infestation: mean = 9.73 ± SD 7.696, median = 7.33, *n* = 16). For provisioning rates, one nest observation from the high infestation group was excluded due to absence of parents during the entire observation. Brood sizes were on average 1.93 ± SE 0.17 (or SD 0.68) for the high infestation intensity nest group (*n* = 16) and 2.33 ± SE 0.24 (or SD 0.70) for the low infestation intensity nest group (*n* = 9). Brood size did not depend significantly on infestation level (glm (quasipoisson family), *t* = 0.298, *p* = 0.768). Additionally, sample size for nestling measurements differed from the sample size for provisioning rates due to limited access to higher nests to measure nestlings.

All analyses were conducted in R Studio (R Core Team 2021) using the *lme4* and *cars* packages (Bates et al. 2015; Fox and Weisberg 2019). We conducted separate generalized linear models (GLM) of the Poisson family with provisioning rates as the response variables. A separate GLM of the quasipoisson family was done for female provisioning rates with significant (*p* = 0.024) underdispersion. Predictors for each model included infestation group (high and low infestation), the number of nestlings per nest on observation day, and the time each observation started between 6:30 and 9:00 a.m. For the duration of female nest attention (min per hour), we conducted a linear model (LM) with infestation group, number of nestlings on observation day and time of day at start of observation as predictors. Tarsus length (mm) and body mass (g) of nestlings were analyzed using linear mixed models (LMM) with the infestation group and number of nestlings per nest on the date of measurements as predictors. Nest ID was included in each LMM as a random factor, to control for pseudoreplication since multiple nestlings were measured from a nest. Each model was checked for normal distribution of residuals using tests for normality and by visually inspecting the QQ plots. Each GLM was additionally tested for overdispersion and heteroscedasticity, while LMMs were checked for heteroscedasticity only. No problems with dispersion or heteroscedasticity were detected, except for the underdispersion in the female provisioning rates model, addressed using the quasipoisson family. For all tests, statistical significance was considered as *p* ≤ 0.05.

## Results

### Food provisioning and breeding success

The total food provisioning rate per nest was not significantly affected by infestation level with *P*. *downsi* (Tables 1, 2), nor the number of nestlings or time of observation (Table 2). There was no significant difference in male provisioning rate between nests with low or high infestation level or the number of nestlings (Table 2). Male provisioning rates were close to significantly higher earlier in the mornings (*p* = 0.056; Table 2). Female provisioning rate was significantly lower at nests with higher infestation, but not significantly affected by the number of nestlings or time of observation (Table 2). Although females spent on average more time attending nestlings of the high infestation group, the difference was not significant, nor affected by brood size (Table 2). Female nest attendance was significantly higher earlier in the morning than later (Table 2).

**Table 1:** Food provisioning data for both high and low infestation groups of Green Warbler-Finch nests. Provisioning data are reported as mean values with standard error

	High infestation (*n*)	Low infestation (*n*)
Total provisioning rate	4.88 ± 0.50 (16)	6.22 ± 0.92 (9)
Male provisioning rate	2.13 ± 0.39 (16)	2.11 ± 0.65 (9)
Female provisioning rate	2.75 ± 0.25 (16)	4.11 ± 0.51 (9)
Female nest attention	19.50 ± 3.3 (16)	12.11 ± 4.67 (9)

Number of nests are reported in parentheses after each value. Total per nest, male, and female provisioning rates are reported as mean visits per hour; while the female nest attention is reported as total number of min. per hour the female was sitting in the nest

**Table 2:** Results from mixed models for each dependent variable

Dependent variable	Fixed effect	Model estimate ± SE	*z*-score or *t* value	*p* value
Total provisioning rate	Intercept	2.80 ± 1.192	2.35	0.019
	Infestation level (low)	0.18 ± 0.182	0.97	0.330
	Number nestlings	0.09 ± 0.131	0.70	0.482
	Time of day	− 0.19 ± 0.151	− 1.25	0.210
Male provisioning rate	Intercept	4.29 ± 2.001	2.15	0.032
	Infestation level (low)	− 0.10 ± 0.296	− 0.33	0.739
	Number nestlings	0.04 ± 0.205	0.18	0.854
	Time of day	− 0.49 ± 0.257	− 1.91	0.056
Female provisioning rate	Intercept	0.85 ± 0.993	0.86	0.400
	Infestation level (low)	0.35 ± 0.156	2.26	0.035
	Number nestlings	0.12 ± 0.115	1.08	0.294
	Time of day	− 0.01 ± 0.124	− 0.09	0.928
Female nest attention	Intercept	98.61 ± 32.924	3.00 (*t*)	0.007
	Infestation level (low)	− 6.60 ± 5.386	− 1.23 (*t*)	0.234
	Number nestlings	− 5.79 ± 3.799	− 1.53 (*t*)	0.142
	Time of day	− 9.12 ± 4.127	− 2.21 (*t*)	0.038

Values are the model (LM or GLM) estimate with the standard error, *z*-score or *t*-statistic (specified in parentheses) and *p* value

At low infestation levels, parents were slightly more successful in fledging at least one offspring (78% of nests) compared to high infestation levels (68% of nests), yet the difference was not significant (Mann Whitney *U* test, *W* = 65, *p* = 0.66, *n* = 25).

### Nestling size

Mean nestling mass (g) at age 5 to 6 days old was significantly higher in nestlings with low infestation, compared to nestlings with high infestation (Tables 3, 4). Mean tarsus length (mm) was longer at low infestation levels; however, not significantly so (Tables 3, 4).

**Table 3:** Nestling growth measurements by infestation level, reported as mean per nest with standard error and number of nests in parentheses

	High infestation (*n*)	Low infestation (*n*)
Number of parasites		
Mean per nest	14.36 ± 1.515 (11)	1.7 ± 0.448 (10)
Measurements		
Body mass (g)	5.14 ± 0.236 (11)	6.26 ± 0.183 (10)
Tarsus (mm)	13.20 ± 0.407 (11)	14.50 ± 0.354 (10)

**Table 4:** Results from a linear mixed model with tarsus length and mass as the dependent variables, infestation level as the predictor and nest ID as a random factor to control for pseudoreplication

Dependent variable	Fixed effect	Model estimate ± SE	*t* value	*p* value
Mass	Intercept	4.97 ± 0.552	8.99	< 0.001
	Infestation level (low)	0.95 ± 0.376	2.51	0.023
	Number nestlings	0.12 ± 0.246	0.50	0.622
Tarsus length	Intercept	12.60 ± 0.97	12.99	< 0.001
	Infestation level (low)	1.14 ± 0.654	1.75	0.099
	Number nestlings	0.31 ± 0.430	0.72	0.478

## Discussion

Overall, we found no significant difference between parental provisioning rate in nests with high versus low infestation intensity, similar to other studies on parental food compensation in Darwin finches with *P*. *downsi* nest parasitism, including the Medium Ground Finch (Knutie et al. 2016), Small Tree Finch (Heyer et al. 2021), and Small Ground Finch (O’Connor et al. 2014). Contrastingly, in one study, Galapagos Mockingbirds responded to *P*. *downsi* parasitism with increased provisioning rates (Knutie et al. 2016). Studies on similar systems in North America involving hematophagous larvae of *Protocalliphora* spp. also failed to show a significant difference in parental food provisioning in relation to infestation, as for example, Eastern Bluebirds (*Sialia sialis*) infested by the blowfly *Protocalliphora sialia* (Grab et al. 2019), or House Wrens (*Troglodytes aedon*) infested by *Protocalliphora parorum* (Morrison and Johnson 2002).

Many factors may influence parental food provisioning behavior in Green Warbler-Finches. Proximate factors include nestling begging rates, as demonstrated in Galapagos Mockingbirds (Knutie et al. 2016), food availability, food quality and others. The soil of the typical breeding habitats of the Green Warbler-Finch on Galapagos is often densely covered by invasive plant species (e.g., invasive blackberry) that prevent foraging on or near the ground, thus causing limited access to food that may prevent higher food provisioning rates to nestlings. It is unlikely that parents of highly infested nests brought higher quality food to the nestlings to compensate for parasites, given that nestlings from highly infested nests showed significantly lower body mass than nestlings from nests with low infestation intensity, despite similar food provisioning rates in the two groups of high and low infestation. As a proximate factor, it may be that nestlings in nests with high infestation intensity were too weak to beg, as reported in the Small Ground Finch (O’Connor et al. 2014). However, begging rates of nestlings are rarely assessed in relation to *Philornis* parasitism on Galapagos and elsewhere. Overall, infestation intensity and prevalence were lower in the year of this study than in previous years (Cimadom et al. 2014; Cimadom and Tebbich 2021), potentially due to a weak La Niña event during the breeding season in 2021 (NOAA 2021), which may have led to a lower abundance of insects, including *P*. *downsi*. Although unlikely, infestation rates even in the ‘high parasitism’ nests may have been too low for detecting a significant increase in parental provisioning rates.

Ultimate factors are typically based on life-history trade-offs that will determine the evolution and expression of parental compensation strategies. That is, an increase in food provisioning rates, and thus parental effort, will entail a decrease in residual reproductive value. Thus, the cost of an increase in parental provisioning rates under low food availability, lower food quality, higher predation exposure and many other factors is predicted to ultimately affect future reproduction. It will influence the decision of parents to increase parental effort in a currently infested nest. In an experimental study on Great Tits (*Parus major*), a forced increase in male and female current *reproductive* effort led to an increase in malaria infections (Richner et al. 1995; Oppliger et al. 1996) and affected return rates of breeders the following year (Richner et al. 1995), i.e., future reproduction. In Blue Tits (*Parus caeruleus*), parents of nests experimentally parasitized with the Hen Flea (*Ceratophyllus gallinae*) had lower return rates and breeding success the year following infestation (Richner and Tripet 1999). Similarly, Alpine Swifts (*Apus melba*) experimentally infested with louse flies (*Crataerina melbae*) experienced significantly reduced breeding success the year following infestation (Bize et al. 2004).

Females spent more time brooding the nestlings earlier in the morning while males provisioned food at a close to significantly higher rate earlier in the morning than later on. Nestling thermoregulation at an age of 5–6 days is not yet well developed, and at our study site in the highlands of Santa Cruz, the mornings can be cooler, between ~ 19 and 21 °C, with the temperature typically increasing between sunrise and midday. Thus, earlier in the morning it was likely more profitable for the female to keep nestlings warm and at an optimal temperature for metabolizing food, rather than spending time foraging. Males then partly compensated by higher food provisioning rates earlier in the morning. Similarly, Small Tree Finch females of infested nests spent more time at the nest than females of nests with low parasite numbers (Heyer et al. 2021). It is unknown whether the additional time was used for active brood care or simply due to the weakening of the female, given that parasites also use brooding females as a food source.

The parental food compensation hypothesis predicts that parents will increase investment in nestlings when parasitized, and in turn parasitized nestlings will benefit and show similar growth rates as uninfested ones. Our results lend no support to this hypothesis: nestlings of highly infested nests had poorer growth and female parents, opposed to the prediction, lowered provisioning rates when highly infested. Lower nestling mass as a consequence of *P*. *downsi* infestation was also reported in the Galapagos Mockingbird, Medium Ground Finch and Small Ground Finch (Fessl et al. 2006a; Koop et al. 2011; Knutie et al. 2016), but interestingly not in another study on the Small Ground Finch (O’Connor et al. 2014) where nestlings of infested and uninfested broods were of similar body size and mass. However, there, nestlings died sequentially in infested nests but survived in nests treated against parasites (O’Connor et al. 2014), which suggests that body size and mass could be maintained due to smaller brood sizes in infested nests. In another similar host-parasite system with *Protocalliphora* parasites, Blue Tit nestlings had higher body mass in nests with lower infestation intensities (Bańbura et al. 2004). In an experimental system with Great Tits infested by Hen Fleas, parents increased provisioning rates, yet nestlings still had lower body mass (Christe et al. 1996). This actually supports theoretical models that predict partial, but not full compensation due to the life-history trade-off in current versus future investment (Perrin et al. 1996). The observed difference in compensation behaviors between non-tropical Passerines and Galapagos landbirds may be explained by the difference in longevity: Galapagos landbirds experience typically long average life span, most likely selected by low predation risk combined with a fluctuating food supply among breeding seasons (Grant and Grant 2011). Thus, their life-history trade-offs are strongly in favor of residual reproductive value and will thus disfavor a significant increase in current reproductive effort and hence the evolution of parental compensation strategies. It may well explain why essentially all studies on Darwin finches so far did not find parental compensation for the effects of nest parasitism by *P*. *downsi*. Thus, future conservation efforts should rather not build on expectations of parental compensation behaviors for the effects of *P. downsi* parasites.

## Data Availability

Datasets generated and analyzed in this study can be provided by the corresponding author upon request.
